# Drivers of a habitat shift by critically endangered Siberian cranes: Evidence from long‐term data

**DOI:** 10.1002/ece3.6720

**Published:** 2020-09-01

**Authors:** Jinjin Hou, Yifei Liu, James D. Fraser, Lei Li, Bin Zhao, Zhichun Lan, Jiefeng Jin, Guanhua Liu, Nianhua Dai, Wenjuan Wang

**Affiliations:** ^1^ Jiangxi Province Key Laboratory of Watershed Ecosystem Change and Biodiversity Center for Watershed Ecology, Institute of Life Science and School of Life Science Nanchang University Nanchang China; ^2^ Ministry of Education Key Laboratory for Biodiversity Science and Ecological Engineering, Coastal Ecosystems Research Station of the Yangtze River Estuary, and Shanghai Institute of EcoChongming (SIEC) Fudan University Shanghai China; ^3^ Department of Fish and Wildlife Conservation Virginia Tech University Blacksburg VA USA; ^4^ Ministry of Education Key Laboratory of Poyang Lake Environment and Resource Utilization Nanchang University Nanchang China; ^5^ Jiangxi Poyang Lake Wetland Conservation and Restoration National Permanent Scientific Research Base National Ecosystem Research Station of Jiangxi Poyang Lake Wetland Nanchang China; ^6^ International Crane Foundation Baraboo WI USA; ^7^ Jiangxi Poyang Lake National Nature Reserve Authority Nanchang China; ^8^ The Institute of Biology and Resources Jiangxi Academy of Sciences Nanchang China

**Keywords:** agricultural habitats, food shortage, *Leucogeranus leucogeranus*, submerged plants, Yangtze River

## Abstract

Many waterbird populations have become increasingly dependent on agricultural habitats for feeding. While habitat destruction has been proposed as a key reason forcing waterbirds to move from natural habitats to agricultural habitats, few have used long‐term data to test this hypothesis. The Siberian crane (*Leucogeranus leucogeranus*) is an IUCN Critically Endangered species. About 98% of its global population winters at Poyang Lake, China. Recently, many cranes shifted from feeding in natural wetlands to agricultural habitats. Here, we integrate bird surveys, *Vallisneria* tuber (the traditional food of cranes in natural wetlands) surveys, water level data, and remotely sensed images from 1999 to 2016 to explore the drivers of this habitat shift. Changes in Siberian crane numbers in natural wetlands and agricultural fields indicated that the habitat shift occurred in the winters of 2015–2016. Analyses using generalized linear mixed models suggested that crane numbers in natural wetlands were positively related to tuber density and the interaction between dry season (October–March) water level and tuber density. The changes in tuber density and dry season water level in 2015–2016 indicated that tuber disappearance may have been the primary driver of the habitat shift, with a smaller effect of high water level. Submerged plants at Poyang Lake have degraded seriously in the past two decades. The plant degradation at Shahu Lake, a sublake of Poyang Lake, may have been caused by high spring water, high winter temperature, and low summer temperature. However, the drivers of tuber disappearance at Poyang Lake may not be restricted to these variables. Because Poyang Lake is an important refuge for many waterbirds in the Yangtze River floodplain, it is urgent to take effective measures to restore its submerged plants and ecosystem health. Agricultural fields can be important refuges for Siberian cranes, mitigating the negative impacts of wetland deterioration.

## Graphical abstract

Our study, using field surveys and remotely sensed images from 18 years, suggests that habitat degradation affecting food shortage may have been the primary driver of the Siberian Crane, an IUCN Critically Endangered species, shifting from feeding in natural wetlands to agricultural habitats at its most important wintering ground, Poyang Lake, China, in the winters of 2015–2016.

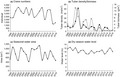

## 1 INTRODUCTION

Many waterbird populations have increasingly become dependent on agricultural habitats for foraging during wintering and migration seasons (Amano, 2009; Bellio, Kingsford, & Kotagama, 2009; Czech & Parsons, 2002). While wetland loss and deterioration have been proposed drivers of these changes (Alonso, Alonso, & Bautista, 1994; Smart & Gill, 2003; Yasué, Patterson, & Dearden, 2007), few have used long‐term monitoring data to test this hypothesis. Alternatively, habitat shifts may be the result of the discovery of more profitable resources. For example, many goose (Fox & Abraham, 2017; Fox et al., 2005), crane (Amano, 2009; Nowald, 2012), heron (Fasola, Rubolini, Merli, Boncompagni, & Bressan, 2010), and shorebird (Toral & Figuerola, 2010) populations have obtained increased food quality and intake rate by shifting to agricultural habitats and have experienced improved breeding success and population increases as a result. Exploring the drivers of foraging habitat shifts is critical to understanding the response of waterbirds to land‐use changes and habitat destruction, and to developing protection measures.

**FIGURE 1:**
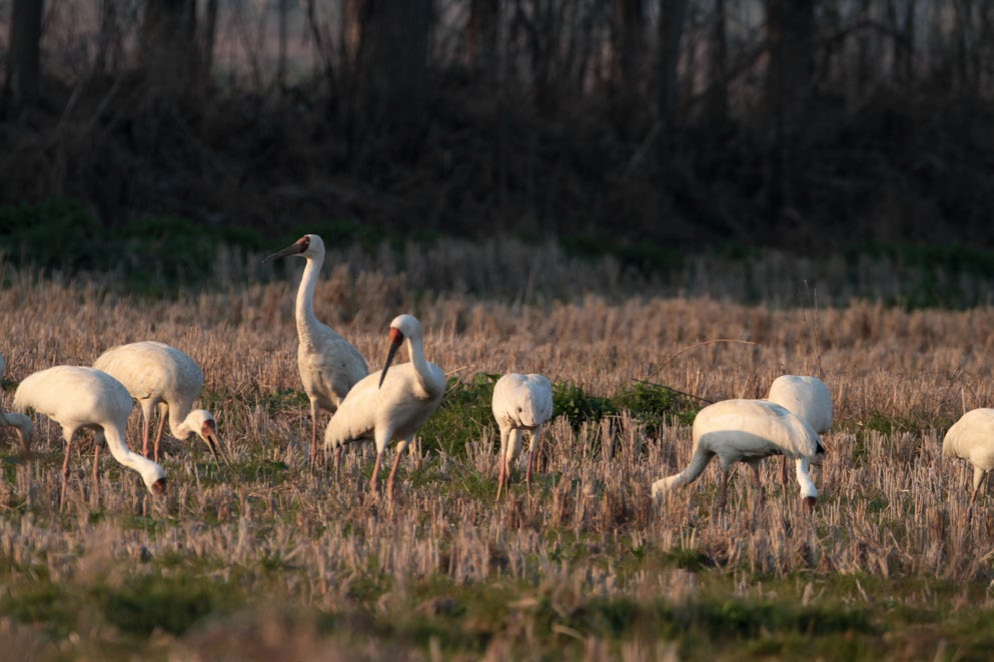
Siberian cranes fed in rice paddies. The photo was taken by Lanhua Wang at Chengxin Farmland, Nanchang City, on 12 February 2017

**FIGURE 2:**
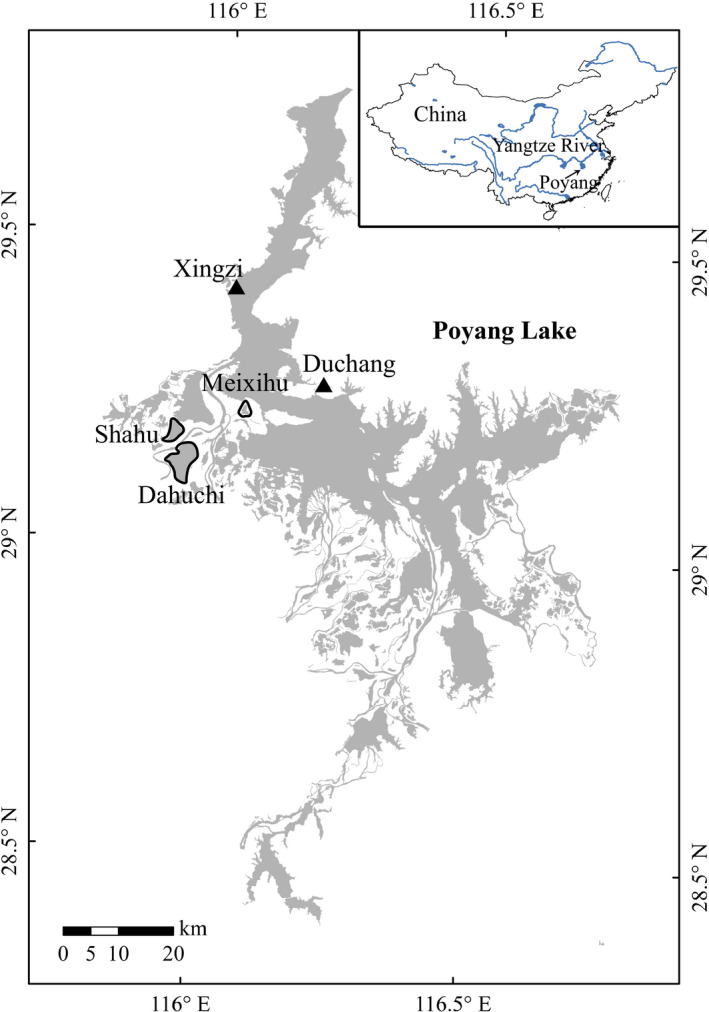
Map of Poyang Lake, China, and the three tuber survey sublakes (Shahu Lake, Dahuchi Lake, and Meixihu Lake). The triangles indicate the Xingzi Hydrological Station and the Duchang Weather Station

The Siberian crane (*Leucogeranus leucogeranus*; Figure 1) is a Critically Endangered species (IUCN, 2018). All three subpopulations of this species breed in Russia, but they winter separately in Iran (western population), India (central population), and China (eastern population; Meine & Archibald, 1996). The populations that winter in Iran and India have declined to fewer than 10 birds (Kanai et al., 2002). The population that winters in China is estimated to comprise 3,800–4,000 individuals (Li, Wu, Harris, & Burnham, 2012). Cranes in the eastern population breed in Yakutia, Siberia, and migrate 5,000 km across eastern China to winter at Poyang Lake, China (Figure 2; Kanai et al., 2002).

The Siberian crane is the most aquatic of cranes, using wetlands for nesting, feeding and roosting (Harris & Mirande, 2013; Meine & Archibald, 1996). At Poyang Lake, the Siberian crane typically feeds in waters <45 cm deep (Wu, Li, & Burnham, 2013; Zhang et al., 2014), where they probe for tubers of submerged plants, primarily *Vallisneria* spp. (*V. spinulosa* and *V. natans*; Wu & Ji, 2002). However, in the winter of 2010 (2010/2011), large numbers of cranes were observed, for the first time, foraging in grasslands on the roots of *Potentilla limprichtii* and bulbs of *Tulipa edulis* (Burnham et al., 2017; Jia et al., 2013). A flood‐induced food collapse was proposed to be the main driver of the habitat and diet shift (Burnham et al., 2017; Jia et al., 2013). The diet shift may have influenced the Siberian crane behavior and energy stores, possibly resulting in lower breeding success in the next breeding season (Burnham et al., 2017). Siberian cranes at Poyang Lake largely returned to shallow waters in the next winter when tuber abundance rebounded (Burnham et al., 2017). However, in recent years, thousands of cranes were observed feed in agricultural habitats including rice paddies and lotus ponds (Lei, 2018; Li, 2018; Wang, Wang, & Hou, 2019). Their main foods changed from *Vallisneria* tubers to rice grains and lotus rhizomes (Hou, Wang, Jin, Wang, & Wang, 2019). Until now, the drivers of this habitat shift have not been explored.

Food abundance is a key factor determining foraging site selection of waterbirds, particularly during the winter (Newton, 1980; Tinkler, Montgomery, & Elwood, 2009; Wang, Fox, Cong, & Cao, 2013). In general, the distribution of animals can reveal information about food distribution, abundance, and availability (Fretwell, 1972). Water depth also is an important factor affecting the use of wetland habitats by waterbirds (Faragó & Hangya, 2012; Ma, Cai, Li, & Chen, 2010). It directly determines the food accessibility for waterbirds because of the restrictions of bird morphology, such as the lengths of tarsometatarsi and necks. Areas of suitable habitats also influence waterbird distribution and abundance, with large areas normally supporting more waterbirds than smaller areas (Li, Yang, et al., 2019; Roshier, Robertson, & Kingsford, 2002).

In this study, we integrated bird surveys, *Vallisneria* tuber surveys, water level data, and remotely sensed images from 1999 to 2016 to examine the drivers of the Siberian crane's shift to agricultural habitats. We first explored the numerical changes of Siberian cranes in natural wetlands to find out when the habitat shift occurred. Then, we examined factors that might have driven the habitat shift. We focused on four variables related to Siberian crane foraging habitat selection: tuber density, tuber biomass, dry season (October–March) water level, and seasonal water area. We defined seasonal water area as the area (km^2^) of Poyang Lake that was exposed during the dry season. It is the difference between the maximum area of the lake during the dry season and permanent water area. Water in seasonal water areas recedes slowly, providing large and dynamic areas of shallow water and mudflats suitable for Siberian cranes. Thus, seasonal water area is an index of foraging habitat available to cranes. We suggest that a variable may have been the driver of the habitat shift (a) if it is an important factor determining the abundance of Siberian cranes in natural wetlands, and (b) if it changed substantially when the habitat shift occurred in a way that might have negatively affected crane foraging (e.g., abnormally low tuber density or biomass). We used generalized linear mixed models (GLMM) to evaluate the effects of the four variables on the numerical change of crane numbers. Because the data were consistent with a tuber collapse being the primary driver of the habitat shift, we further explored whether the tuber collapse may have been driven by abnormal water levels or temperatures, important factors influencing the growth and production of *Vallisneria* (Cao, Li, & Jeppesen, 2014; Xu et al., 2016; Zhao et al., 2017).

## 2 METHODS

### 2.1 Study site

Poyang Lake (28°25′–29°45′N, 115°48′–116°44′E; Figure 2), the largest freshwater lake in China, is one of only two lakes that still connect freely with the Yangtze River. The northern portion of the lake is characterized by a narrow and deep outlet into the Yangtze River and the southern portion by a broader and shallower main extent of lake with delta from 5 tributary rivers (Ganjiang, Fuhe, Xiushui, Xinjiang, and Raohe). Poyang Lake is subject to substantial intra‐annual hydrological fluctuations (average 9.24 m between wet season high and dry season low water levels; Min, 1995). During the wet season (April–September), the inundated area is >2,500 km^2^ and sometimes exceeds 3,000 km^2^ (Feng et al., 2012). During the dry season, the inundated area can shrink to <1,000 km^2^ (Feng et al., 2012). Poyang Lake also is subject to substantial interannual hydrological fluctuations, with the monthly maximum/minimum inundation area ratios between 1.46 and 4.03 in 2000–2010 (Feng et al., 2012). The large hydrological fluctuations are driven primarily by local precipitation, which varies widely year‐to‐year, making Poyang Lake one of the most frequently flooded and also most frequently drought‐stricken areas in China (Min, 2007; Shankman, Keim, & Song, 2006). The water level fluctuation is a key reason that Poyang Lake is among the most productive lakes for waterbirds in the world (Barter, Cao, Chen, & Lei, 2005; Barzen, 2012; Wang, Fraser, & Chen, 2019). The lake contains 102 sublakes, which are inundated and integrated with the main body of Poyang Lake during the wet season, and become isolated during the dry season (Hu, Zhang, Liu, Ji, & Ge, 2015). When sublakes are connected with the main body of Poyang Lake, their water levels are the same as the main body. When sublakes are isolated, their water levels are primarily controlled by local fishermen who manage water levels with sluices (Wang, Fraser, et al., 2019). Fishermen release water when they want to catch fish. As water levels recede, extensive wet meadows, shallow waters and mudflats are exposed, providing a wide range of foraging and roosting habitat for hundreds of thousands of migratory waterbirds.

### 2.2 Data collection

We collected data on Siberian Crane numbers in natural wetlands of Poyang Lake for the winters of 1999 to 2016 from the literature (Table S1). Crane surveys were conducted by the Poyang Lake National Nature Reserve (PLNNR) and the Jiangxi Wildlife Management Bureau (JWMB). The PLNNR and the JWMB usually conducted synchronous ground surveys, where field staff observed and counted Siberian cranes at approximately the same time on the same date, to avoid double counting or missing birds. Depending on lake size, 1–3 fixed observation points were established at each sublake. The observation sites were located on high ground so that most birds could be observed and counted. Although there were some differences in survey areas between the censuses conducted by the PLNNR and the JWMB, both censuses included more than 68 sublakes, covering most of the areas used by Siberian cranes (Li et al., 2012; Li, Qian, Silbernagel, & Larson, 2019). Therefore, these data are comparable and could be integrated to reflect population dynamics of Siberian cranes. Most surveys were conducted in December and January, when crane numbers were stable. The PLNNR conducted 1–2 census, and the JWMB conducted one census each winter. We used the average number counted each winter in our analyses.

In collaboration with the International Crane Foundation, the PLNNR has monitored *Vallisneria* tuber density and biomass at three sublakes of Poyang (Dahuchi Lake, Shahu Lake, and Meixihu Lake; Figure 2) since the winter of 1999 (Wu, Li, Liu, & Zeng, 2012). We obtained the tuber survey results for the winters of 1999–2016 from the literature (Table S2). The three sublakes were selected based on size, human disturbance, and environmental conditions (Wu et al., 2012). Dahuchi Lake and Shahu Lake are extensively used by Siberian cranes (Li et al., 2012; Wu et al., 2013). As tubers do not grow after October, tuber surveys usually were conducted in November, rarely in October and December (Wu et al., 2013). For each sublake, two crossed sampling transects were selected, one located along the long side of the sublake, and the other located on the short side. Plots were established every 50 m, with four quadrats in each plot. The plots were in the same place each year as determined with GPS locations. Samples were excavated with a locally made steel grab sampler. The sampler has two long handles. At the end of the handles, there are two scoops facing each other. The sampler was inserted into the substrate and the scoops brought together to collect about 15 cm long × 13 cm wide × 20 cm high sample of substrate. The same sampler was used in every survey so that the sample volume was constant. Mud was washed away through a mesh (pore size 0.5 cm × 0.5 cm), and tubers were cleaned and counted. The dry weight of tubers in each quadrat was weighed after oven‐drying at 80 ℃ until constant weight. The tuber density (ind/m^2^) and biomass (g/m^2^) in each plot were calculated by summing the total tuber number or tuber mass across four quadrats and dividing by the sum of the area of the four quadrats.

To explore the impacts of water level on Siberian crane numbers, we obtained daily water levels (Wu Song elevation datum) recorded at the Xingzi Hydrological Station (Figure 2). The hydrological station is located at the deep outlet of Poyang Lake. Its water level data have frequently been used to represent the water level of Poyang Lake (Wang et al., 2013; Xia, Yu, & Fan, 2010). Water level data of Dahuchi Lake, Shahu Lake, and Meixihu Lake (Wu Song elevation datum) also were collected to analyze the influences of water level on tuber abundance. Water level data of sublakes were used because tuber surveys were conducted at sublakes. During the dry season, water levels of most sublakes are controlled by local fishermen through sluices. Therefore, their water levels are independent from the water level of the main body of Poyang Lake. We also collected monthly air temperature data recorded at the Duchang Weather Station (Figure 2) to explore the influences of temperature on tuber abundance. The temperature data were obtained from the China Meteorological Administration (http://www.cma.gov.cn/).

### 2.3 Seasonal water area delineation

Due to the strong intra‐annual dynamics of water surface areas, we integrated all the available Landsat 5/7/8 surface reflectance images (30 m spatial resolution) from the Google Earth Engine (GEE) platform during the dry seasons of 1999 to 2016 to capture water body variability (Zou et al., 2017). We removed poor‐quality observations caused by clouds, shadows, and scan‐line gaps, using the Landsat quality assurance (QA) band. This left us with 1,022 usable images (Table S3). We employed three widely used indices to identify the surface water: the Normalized Difference Vegetation Index (NDVI; Tucker, 1979), Enhanced Vegetation Index (EVI; Huete et al., 2002), and modified Normalized Difference Water Index (mNDWI; Xu, 2006). NDVI and EVI are closely related to the greenness of vegetation, and mNDWI is one of the most commonly used indices for mapping surface water bodies (Wang et al., 2020). The three indices were calculated based on reflectance of different wavelengths measured in the Landsat images on a per pixel basis. We used a mNDWI/VIs algorithm combining NDVI, EVI, and mNDWI to reduce commission error in water detection (Xiao, Yu, & Wu, 2007; Xiao et al., 2005; Zou et al., 2017). Pixels whose water signal were stronger than their vegetation signal (mNDWI > EVI or mNDWI > NDVI) were classified as water pixels. To further remove the vegetation noise, EVI < 0.1 was used to remove the mixed pixels of water and vegetation. Therefore, only those pixels that met the criteria ((mNDWI > EVI or mNDWI > NDVI) and (EVI < 0.1)) were classified as surface water body pixels while the rest were classified as nonwater pixels (Zou et al., 2017, 2018). For each pixel, we calculated the water frequency, which was the ratio of the number of observations identified as water to the total number of useable observations within a dry season (Zou et al., 2017). Pixels with a water frequency ≥ 0.25 were classified as water pixels (Zou et al., 2017). Water pixels with a water frequency ≥ 0.75 were classified as permanent water pixels since they have water most of the dry season (Zou et al., 2017). For each winter, we generated maps of maximum water bodies (water frequency ≥ 0.25) and permanent water bodies (water frequency ≥ 0.75), respectively, and then calculated their areas. Area of annual seasonal water bodies was calculated as the area of maximum water bodies minus the area of permanent water bodies.

### 2.4 Statistical analyses

To explore the drivers of the cranes’ habitat shift, four variables were used: tuber density, tuber biomass, dry season water level, and seasonal water area. Tuber density and biomass were the average values of Dahuchi Lake, Shahu Lake, and Meixihu Lake. Dry season water level was the average water level recorded at the Xingzi Hydrological Station between October and March. Annual data of the four variables and crane numbers in the winters of 1999–2016 were used. GLMMs were used to evaluate the effects of the four explanatory variables on crane numbers. Before building the GLMMs, we assessed multicollinearity among explanatory variables by examining the variance inflation factors (VIFs). All VIF values were less than 2.5, indicating little evidence for multicollinearity (O'brien, 2007). All explanatory variables were standardized to a mean of 0 and standard deviation of 1 prior to analysis to facilitate comparison of coefficients among models. We used the four explanatory variables and two interactions (dry season water level × tuber density, dry season water level × tuber biomass) as fixed effects. Year was included as a random effect. The GLMM analysis generated a complete set of models with all possible combinations of all explanatory variables (i.e., 64 models) and graded the models according to their Akaike's information criterion corrected for small sample sizes (AIC*c*) and Akaike weights (Burnham & Anderson, 2002). Following Burnham and Anderson (2002), we selected models with ΔAIC*c* < 2 (the difference between each model's AIC*c* and the lowest AIC*c*) as candidates for top models. Model averaging was subsequently applied to estimate the correlation coefficient (β) and their 95% confidence intervals (CI) for each variable in the top models (Burnham & Anderson, 2002). The model averaging calculation was done on the top models. Model averaging incorporates the uncertainty associated with model selection by combining estimates over a set of models (Schomaker & Heumann, 2014). The relative importance of each variable was estimated by summing the Akaike weights across all models where a variable was included (Burnham & Anderson, 2002). We used relative importance and 95% CI of coefficient to determine the relative effect of each variable (Arnold, 2010; Catlin, Fraser, & Felio, 2015). All statistical analyses were conducted in R 3.6.0 with the packages lme4, MuMIn, vegan, and car.

**FIGURE 3:**
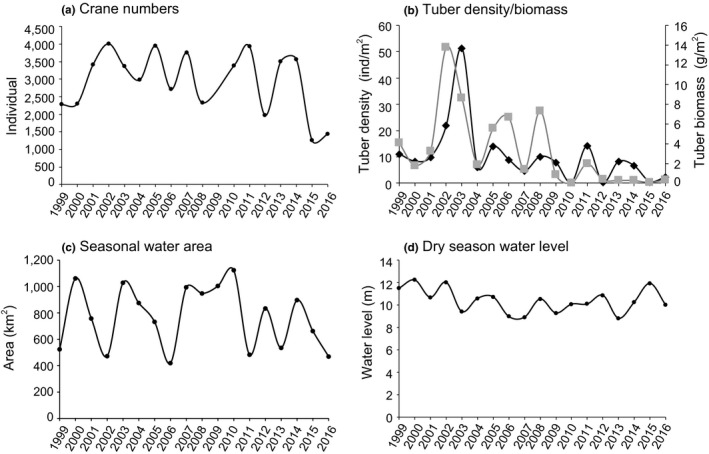
Annual data for (a) Siberian crane numbers in natural wetlands of Poyang Lake. (b) Average tuber density (ind/m^2^; black line) and biomass (g/m^2^; gray line) at Dahuchi Lake, Shahu Lake, and Meixihu Lake. (c) Seasonal water area (km^2^) during the dry season. (d) Dry season (October–March) water level (m) at the Xingzi Hydrological Station

As the graphs of crane number, tuber density, tuber biomass, dry season water level, and seasonal water area look cyclical (Figure 3), we used Fourier analysis, which has been previously used to detect population cycles (Blomqvist, Holmgren, Åkesson, Hedenström, & Pettersson, 2002; Fraser, Karpanty, Cohen, & Truitt, 2013) to examine the cycles in these variables. The Fourier analyses were conducted using SAS 9.4 (SAS Institute, Cary, NC, USA). To explore whether submerged plants decreased in density and biomass over the study period, we calculated Spearman's rho correction coefficients between tuber density and year, and between tuber biomass and year.

To analyze the effects of water level and air temperature on tuber density and biomass, data from Dahuchi Lake and Shahu Lake were considered separately. Meixihu Lake was not included in this analysis because its water level was not recorded after 2010. Daily water level of Dahuchi Lake and Shahu Lake were averaged to derive yearly seasonal measures (December–February: winter; March–May: spring; June–August: summer; September–November: autumn) to avoid multicollinearity between variables. Daily air temperatures recorded at the Duchang Weather Station also were averaged to derive yearly seasonal measures. As *Vallisneria* overwinter in the forms of seeds and tubers which are produced before November (Li, Lan, Chen, & Song, 2018; Yuan, Li, & Li, 2013), winter water level and temperature would most likely affect tuber abundance in the following year. Therefore, eight explanatory variables were used, including winter water levels and temperatures in the preceding year, spring water levels and temperatures, summer water levels and temperatures, and autumn water levels and temperatures. Annual data spanning the winters of 1999–2016 were used. Linear regression models were used to evaluate the effects of the eight variables on tuber density and biomass. The eight variables were entered as independent variables, and tuber density and tuber biomass were entered as response variables. All explanatory variables had VIF values less than 4, suggesting multicollinearity did not affect the models. The linear regression analyses generated a total of 256 models, and models with ΔAIC*c* < 2 were selected as candidates for the top models. Similar to the crane number analyses, the correlation coefficients and their 95% CI’s for each variable in the top models was calculated by model averaging, and the relative importance of each variable was estimated by summing the Akaike weights of the models containing the variable. The relative importance and 95% CI’s of coefficients were used to determine the relative effect of each variable.

## 3 RESULTS

Siberian crane numbers in natural wetlands fluctuated over our study period (mean = 2,942 ± 855 *SD*; Figure 3a and Figure S1). Several large year‐to‐year declines have occurred (e.g., 2012, 2015), with the most severe decline happening in the winters of 2015–2016, from 3,552 individuals in 2014 to 1,247 individuals in 2015 and 1,437 individuals in 2016.

Mean tuber density and tuber biomass were 10.33 ind/m^2^ ± 11.27 *SD* and 3.26 g/m^2^ ± 3.68 *SD*, respectively (Figure 3b). Tuber density (Spearman's *ρ* = −0.59, *p* = .01) and tuber biomass (Spearman's *ρ* = −0.71, *p* = .00) declined significantly over the study period. Tuber density declined to nearly zero in the winters of 2010, 2012, and 2015–2016, and tuber biomass declined to nearly zero in the winters of 2010 and 2012–2016. The average seasonal water area was 767.62 km^2^ ± 230.76 *SD* (Figure 3c and Figure S2), and the average dry season water level was 10.37 m ± 1.03 *SD* (Figure 3d). As the habitat shift of Siberian cranes occurred in the winters of 2015–2016 (see Section 4 for details), we focused on the values in the two winters. The seasonal water area in winter 2015 was similar to the historical average level, while the area in winter 2016 was the second lowest over the study period. The dry season water level in winter 2015 was the third highest over our study period, and the water level in winter 2016 was similar to the historical average water level.

The *p*‐values for the Kolmogorov–Smirnov statistic of crane numbers, tuber density, tuber biomass, seasonal water area, and dry season water level were 0.52, 0.36, 0.07, 0.23, and 0.47, respectively. Thus, there was no evidence of cyclic patterns based on Fourier analysis.

The top two models for crane numbers contained dry season water level, tuber density, seasonal water area, and the interaction between dry season water level and tuber density (Table 1). The 95% CI’s of coefficients for tuber density and the interaction between dry season water level and tuber density did not overlap 0, and the relative importance of the two variables were >0.70 (Table 2). Thus, the two parameters might be closely related to crane numbers in natural wetlands. Both variables were positively correlated with crane numbers (Table 2). Although dry season water level and seasonal water area also were included in the top two models, the 95% CI of coefficients for dry season water level overlapped with 0 and the relative importance value of seasonal water area was only 0.43. Therefore, their effects on crane numbers might be limited.

**TABLE 1:** Top linear regression models (ΔAIC*c* < 2) for Siberian crane numbers in natural wetlands of Poyang Lake, China

ID	Models	*df*	AIC*c*	ΔAIC*c*	ω*_i_*
1	Dry season water level + tuber density + seasonal water area + dry season water level × tuber density	6	282.67	0	0.30
2	Dry season water level + tuber density + dry season water level × tuber density	5	283.13	0.46	0.24

Abbreviations: AIC*c*, Akaike information criterion for small sample size; *df*, degrees of freedom; ΔAIC*c*, the difference between each model's AIC*c* and the lowest AIC*c*; ω*_i_*, Akaike weights.

**TABLE 2:** Correlation coefficient (β), 95% confidence intervals (CI) of coefficient, and relative importance values for the effects of variables on Siberian crane numbers in natural wetlands of Poyang Lake, China

Variable	β	95% CI	Relative importance
Dry season water level	–0.11	–0.23 to 0.00	0.86
Seasonal water area	0.07	0.02 to 0.25	0.43
Tuber density	0.30	0.15 to 0.45	0.82
Dry season water level × tuber density	0.30	0.13 to 0.46	0.73

The top models for tuber density at Shahu Lake contained last winter temperature, spring water level, autumn temperature, and summer water level (Table 3). The 95% CI’s of coefficients for last winter temperature and spring water level did not overlap with 0, and their relative importance values were about 0.60 (Table 4). Thus, the two variables might be closely related to tuber density at Shahu Lake. Both variables were negatively related to tuber density (Table 4). The 95% CI’s of coefficients for autumn temperature and summer water level included 0, and their relative importance values were only about 0.30. Thus, the two variables had little effect on tuber density.

**TABLE 3:** Top linear regression models (ΔAIC*c* < 2) for explaining tuber density and tuber biomass at Shahu Lake, a sublake of Poyang Lake, China

ID	Models	*df*	AIC*c*	ΔAIC*c*	ω*_i_*
Tuber density					
1	Last winter temperature + spring water level	4	126.12	0.00	0.13
2	Last winter temperature + autumn temperature + spring water level	5	127.19	1.07	0.08
3	Last winter temperature + summer water level	4	127.67	1.55	0.06
4	Spring water level	3	127.88	1.76	0.06
Tuber biomass					
1	Last winter temperature + autumn temperature	4	107.16	0.00	0.14
2	Last winter temperature + autumn temperature + summer water level	5	108.90	1.74	0.06
3	Last winter temperature + autumn temperature + spring temperature	5	108.94	1.78	0.06

Abbreviations: AIC*c*, Akaike information criterion for small sample size; *df*, degrees of freedom; ΔAIC*c*, the difference between each model's AIC*c* and the lowest AIC*c*; ω*_i_*, Akaike weights.

**TABLE 4:** Correlation coefficient (β), 95% confidence intervals (CI) of coefficient, and relative importance values for the effects of variables on tuber density and biomass of Shahu Lake, a sublake of Poyang Lake, China

Variable	β	95% CI	Relative importance
Tuber density			
Last winter temperature	–4.26	–8.36 to –0.16	0.61
Autumn temperature	5.66	–1.99 to 13.30	0.30
Spring water level	–10.26	–19.65 to –1.20	0.59
Summer water level	–4.57	–9.25 to 0.12	0.28
Tuber biomass			
Last winter temperature	–2.56	–4.89 to –0.23	0.67
Spring temperature	2.42	–1.35 to 6.18	0.22
Autumn temperature	5.05	0.45 to 9.66	0.70
Summer water level	–1.66	–4.21 to 0.90	0.26

The top models for tuber biomass at Shahu Lake included last winter temperature, autumn temperature, summer water level, and spring temperature (Table 3). The 95% CI’s of coefficients for last winter temperature and autumn temperature did not overlap with 0, and their relative importance values were >0.65 (Table 4). Last winter temperature was negatively related to tuber biomass, and autumn temperature was positively related to tuber biomass (Table 4). Although summer water level and spring temperature also were included in the top models, their 95% CI of coefficients included 0, and their relative importance values were <0.30.

The top models for tuber density and tuber biomass of Dahuchi Lake contained two variables, spring temperature and summer temperature (Table 5). The effects of the two variables on tuber density and tuber biomass of Dahuchi Lake was subtle because their 95% CI of coefficients included 0, and their relative importance values were <0.50 (Table 6).

**TABLE 5:** Top linear regression models (ΔAIC*c* < 2) for tuber density and tuber biomass at Dahuchi Lake, a sublake of Poyang Lake, China

ID	Models	*df*	AIC*c*	ΔAIC*c*	ω*_i_*
Tuber density					
1	Summer temperature	3	169.35	0.00	0.13
2	Intercept	2	169.65	0.30	0.11
3	Summer temperature + spring temperature	4	170.81	1.45	0.06
4	Spring temperature	3	170.96	1.61	0.06
Tuber biomass					
1	Intercept	2	99.02	0.00	0.14
2	Summer temperature	3	99.54	0.52	0.10
3	Spring temperature	3	100.34	1.32	0.07

Abbreviations: AIC*c*, Akaike information criterion for small sample size; *df*, degrees of freedom; ΔAIC*c*, the difference between each model's AIC*c* and the lowest AIC*c*; ω*_i_*, Akaike weights.

**TABLE 6:** Correlation coefficient (β), 95% confidence intervals (CI) of coefficient, and relative importance values for the effects of variables on tuber density and biomass of Dahuchi Lake, a sublake of Poyang Lake

Variable	β	95% CI	Relative importance
Tuber density			
Summer temperature	20.57	–3.81 to 44.95	0.47
Spring temperature	–15.40	–40.92 to 10.13	0.29
Tuber biomass			
Summer temperature	2.26	–0.89 to 5.41	0.37
Spring temperature	–1.93	–5.23 to 1.38	0.29

## 4 DISCUSSION

### 4.1 Habitat shift of Siberian cranes

Long‐term monitoring data indicate that Siberian crane numbers in natural wetlands fluctuated in the winters of 1999–2016. This could have been driven by breeding success variation (Germogenov et al., 2013), weather variation on wintering ground (Li et al., 2014), or bird being missed on surveys due to habitat shifts (Li et al., 2019). The crane numbers underwent several large year‐to‐year declines (e.g., 2012, 2015); however, the decline in the winters of 2015–2016 was more severe than other declines, and concurrently, Siberian cranes at rice paddies and lotus ponds increased. For example, Wuxing Reclamation Farm (Nanchang City), a reclamation area composed of rice paddies and lotus ponds near Poyang Lake, supported an average of 47 (range: 0–170) cranes in the winters of 2010–2014, but the numbers increased to 297 in winter 2015 and 1,300 in winter 2016 (Wang et al., 2019). Lei (2018) indicated that Siberian cranes began to appear at farmlands of Yugan County (Shangrao City) in winter 2015, and more than 1,300 individuals were recorded in winter 2016. Li (2018) conducted waterbird surveys on seven transects at rice paddies adjacent to Poyang Lake, recoding 20 Siberian cranes in winter 2015 and 1,113 cranes in winter 2016. Although crane numbers in natural wetlands declined in other winters (e.g., 2012), no literature reported crane number increase at agricultural fields in these winters, and the local wildlife managers were unaware of any such movements. Cranes moving out of the survey areas might contribute to the decline of counting number in winter 2012 (Liu et al., 2013).

### 4.2 Drivers of the habitat shift

Our GLMM analyses indicate that crane numbers in natural wetlands were positively related to tuber density. Tuber density collapsed in the winters of 2010, 2012, and 2015–2016 at Poyang Lake. Waterbirds are expected to give up a foraging site when food density drops below a threshold value, and this giving‐up density threshold has been reported in many waterbirds (Jonzen, Nolet, Santamaria, & Svensson, 2002; Nolet et al., 2001; Sponberg & Lodge, 2005). The tuber collapse in the winter of 2010 was suggested to be a major driver for Siberian cranes moving from shallow waters to grasslands at Poyang Lake in that winter (Burnham et al., 2017; Jia et al., 2013). The decline of Siberian cranes in natural wetlands in winter 2012 was coincident with a tuber decline. It is likely that the tuber collapse in the winters of 2015–2016 was a major driver for Siberian cranes moving from natural wetlands to agricultural habitats. Similarly, food shortage was thought to be the main driver of other crane (Zheng, Zhou, Zhao, & Xu, 2015), goose (Clausen, Clausen, Fælled, & Mouritsen, 2012), and swan (Nolet, Bevan, Klaassen, Langevoord, & der Heijden, 2002) populations moving from natural wetlands to agricultural habitats.

The interaction between tuber density and dry season water level was an important factor influencing crane numbers in natural wetlands. A flood occurred at Poyang Lake in the winter of 2015 (Zeng, Schmitt, Li, & Zhu, 2017), and water reached the third highest level during our study period. Even when food is abundant, high water can reduce its availability if cranes cannot reach it (Ma et al., 2010), and cranes may leave in search of food elsewhere (Chen et al., 2014; Zhang et al., 2014). The 2015 winter flood at Poyang Lake led to the decline of total waterbird abundance (Wang, Wang, Hou, & Ouyang, 2019), and to diet and foraging habitat shifts of two goose species (Aharon‐Rotman et al., 2017). It likely reduced Siberian crane food availability as they primarily feed in shallow waters and mudflats (Wu et al., 2013; Zhang et al., 2014). Thus, the combination of low tuber density and high water levels probably drove cranes to move to agricultural habitats in winter 2015. The dry season water levels in winter 2016 were similar to the historical average water level, while many Siberian cranes still foraged in agricultural fields. Thus, high water levels may have contributed to the habitat shift, but played a less important role than low tuber density.

Tuber biomass did not closely relate to crane numbers in natural wetlands. Tuber biomass collapsed in the winters of 2012–2016, while Siberian cranes left natural wetlands in the winters of 2015–2016. Thus, many Siberian cranes still searched for tubers in natural wetlands before the winters of 2015, even though there only were small tubers available. Only when tuber density also collapsed, did Siberian cranes give up natural wetlands.

The effect of seasonal water area on Siberian crane numbers in natural wetlands was relatively weak. The seasonal water area in the winter of 2015 was similar to the historical average level, while the area in the winter of 2016 was the second smallest over the study period. There were similar small seasonal water areas in the winters of 2002 and 2006, while most cranes still foraged in natural wetlands, and numerous cranes were not recorded in agricultural fields. Thus, although the foraging habitat may have been diminished in winter 2016, its effect on habitat shift was less than the effect of the tuber collapse and high water level.

### 4.3 Reasons for the tuber collapse

The tuber density and biomass declined significantly at the three sublakes (Dahuchi Lake, Shahu Lake, and Meixihu Lake) of Poyang Lake over the study period, indicating severe degradation of the submerged plants and the wetland ecosystem. Similarly, Hu and Lin (2019) indicated that area of submerged plants declined between 1983 and 2013, from about 1,124 to 700 km^2^ (37.7% decline) throughout Poyang Lake. Submerged plants were widely distributed over Poyang Lake in 1983, but restricted to few sublakes by 2013 (Hu & Lin, 2019). The number of plant species also decreased with the disappearance of some plant species sensitive to water pollution (Hu & Lin, 2019; Jian et al., 2015).

Submerged plant growth is controlled by numerous factors, including water depth, temperature, light, nutrients, substrate, and water movements (Bornette & Puijalon, 2011). Water depth, which substantially affects underwater light availability, is a key factor controlling growth of submerged macrophytes (Søndergaard et al., 2013; Xu et al., 2016). Optimal water depth can promote *Vallisneria* growth (Li et al., 2018; Wei et al., 2018), while excessively high water level inhibits its growth (Xiao et al., 2007; Xu et al., 2016). Our results indicated that tuber density at Shahu Lake was negatively related to spring water level. Spring is the time of the initial growth of *Vallisneria* (Li et al., 2018) and is regarded as a key stage during the annual life history of many aquatic plants (Jia, Cao, Yésou, Huber, & Fox, 2017; Nishihiro & Washitani, 2009; Paillisson & Marion, 2011). High spring water level likely negatively affects tuber germination and shoot development of *Vallisneria* (Xu et al., 2016) and thus negatively affects tuber density in autumn.

Winter temperature influences seed and tuber germination of *Vallisneria* (Kauth & Biber, 2014; Xiao, Xing, & Liu, 2010; Zhao et al., 2017) and other aquatic plants (Schütz, 2000; van Wijk, 1989; Yin et al., 2009) as cold stratification is necessary for dormancy release and spring germination. Seeds and tubers normally have a higher germination rate after a cold storage than after a warm storage (Imanishi & Imanishi, 2014; Moravcová, Zákravský, & Hroudová, 2001). The negative relationships between last winter temperature and *Vallisneria* tuber density and biomass at Shahu Lake are consistent with previous studies. Higher winter temperature in the preceding year may lead to lower germination of *Vallisneria* seeds and tubers and thus lower plant density, which may further lead to lower tuber density and biomass.

In contrast to last winter temperature, summer temperature was positively correlated with tuber biomass at Shahu Lake. Summer is the active growing season of *Vallisneria* plants (Cao et al., 2014). Higher summer temperature can enhance the growth of *Vallisneria* (Bartleson, Hunt, & Doering, 2014; Cao et al., 2014) and other aquatic plants (Barko & Smart, 1981; Rooney & Kalff, 2000), while low summer temperature can impede plant growth.

Thus, high spring water level, high winter temperature, and low summer temperature probably contributed jointly to the tuber disappearance at Shahu Lake. Due to global climate change and human activities, spring water level (Li, Tao, Yao, & Zhang, 2016) and winter temperature (Li et al., 2016; Ye, Zhang, Liu, Li, & Xu, 2013) have increased at Poyang Lake in recent decades. The operation of the Three Gorges Dam, located at the upper reaches of the Yangtze River, has also led to increase of spring water level at Poyang Lake (Wu et al., 2009). In addition, the frequency of extreme precipitation (Sun et al., 2014) and temperature (Wang, Zhang, Wang, Ma, & Sun, 2014) has increased. These climate changes are consistent with the large fluctuation of tuber abundance over our study period and frequent occurrences of tuber disappearances in recent years. Under future climate change, precipitation and temperature extremes are expected to increase in frequency and intensity (Xu, Xu, Gao, & Luo, 2009; Ye, Zhang, Bai, & Hu, 2011). Therefore, tuber disappearance may occur more frequently in the future, potentially threatening the survival of Siberian cranes.

Unlike at Shahu Lake, a close relationship between tuber density/biomass and water level/temperature was not detected at Dahuchi Lake. These contrasting results indicate that the drivers of tuber collapse might be different among different sublakes. Besides water level and temperature, other factors might also have contributed to the tuber collapse at Poyang Lake. Some potential drivers include declining water quality (Li et al., 2015; Wang, Liu, Fang, & Feng, 2014), extensive aquaculture of fish and crabs (Wang, Fraser, & Chen, 2017), and herbivory by the invasive crayfish (*Procambarus clarkia*; Carreira, Dias, & Rebelo, 2014; Zou et al., 2014). In addition, the operation of the Three Gorges Dam and widespread sand dredging, leading to inundation shrinkage of Poyang Lake during the dry season, have compressed the living space of submerged plants (Han, Feng, Hu, & Chen, 2018; Lai, Jiang, Yang, & Lu, 2014).

### 4.4 The role of agricultural fields in Siberian crane protection

Siberian cranes foraged in agricultural fields when tubers collapsed in natural wetlands in the winters of 2015–2016. This indicates that agricultural fields can be important refuges for Siberian cranes, mitigating the negative impacts of wetland deterioration. Human activity in agricultural fields in eastern China is considerably higher than in many countries (Yu et al., 2017; Zhao, Wang, Cao, & Fox, 2018). Approximately 10 million people live around Poyang Lake, and more than 14 million free‐ranging domestic fowl are fed on spilled grain in rice paddies (Choi et al., 2016; Xing & Wang, 2016). Such intensive free‐range poultry is extremely rare in other countries (Zhao et al., 2018). Intensive gleaning by domestic poultry and associated human presence may affect foraging efficiency of Siberian cranes (Zhao et al., 2018). High disturbance may be why Chinese wintering geese continue to use natural wetlands and fail to exploit the riches of the modern agricultural habitats (Yu et al., 2017; Zhao et al., 2018). Because of high human disturbance, Siberian cranes spent twice as much time alert in agricultural fields (25.02%) than in natural wetlands (11.94%; Shao et al., 2018). As foraging habitat quality greatly influences waterbird behavior, energy accumulation, breeding success, and population dynamics (Burnham et al., 2017; Newton, 2004; Tinkler et al., 2009), research should focus on the impacts of agricultural feeding on Siberian Crane fitness to provide a scientific underpinning for conservation.

## 5 CONCLUDING REMARKS

In conclusion, our study, using long‐term data, suggests that Siberian cranes shifted foraging habitats from natural wetlands to agricultural fields in the winters of 2015–2016. Tuber disappearance was likely a key driver of this shift. High dry season water levels might also have contributed to the habitat shift, but to a less extent than tuber disappearance. The submerged plants at Poyang Lake have degraded seriously in the past two decades. The plant degradation at Shahu Lake was likely driven by high spring water level, high winter temperature, and low summer temperature. The subtle effects of water level and temperature on tuber abundance at Dahuchi Lake indicated that, besides water level and temperature, other factors might also have contributed to the submerged plant degradation at Poyang Lake. Because Poyang Lake is an important refuge for many waterbirds in the Yangtze River floodplain (Wang et al., 2017), effective conservation measures are needed to restore its ecosystem health. Agricultural fields are important refuges of Siberian cranes, buffering the negative impacts of wetland degradation. However, because the human disturbance in agricultural fields is high, further research is needed to evaluate the impacts of agricultural feeding on Siberian Crane fitness.

## CONFLICT OF INTEREST

None declared.

## AUTHOR CONTRIBUTIONS

**Jinjin Hou:** Data curation (equal); Writing‐original draft (equal). **Yifei Liu:** Formal analysis (equal); Writing‐original draft (equal). **James D. Fraser:** Conceptualization (equal); Writing‐review & editing (equal). **Lei Li:** Data curation (equal); Investigation (equal); Writing‐review & editing (equal). **Bin Zhao:** Writing‐review & editing (equal). **Zhichun Lan:** Formal analysis (equal); Writing‐review & editing (equal). **Jiefeng Jin:** Investigation (equal). **Guanhua Liu:** Investigation (equal). **Nianhua Dai:** Writing‐review & editing (equal). **Wenjuan Wang:** Conceptualization (equal); Funding acquisition (equal); Visualization (equal); Writing‐original draft (equal).

## Supporting information

Supplementary Material

## Data Availability

All data used in this paper are included in the manuscript.
