# Supplementary material for: Drivers of a habitat shift by critically endangered Siberian cranes: Evidence from long‐term data
Source: Ecol Evol. 2020 Sep 1;10(20):11055–68. doi: 10.1002/ece3.6720 (PMC7593143; doi:10.1002/ece3.6720)

**Appendices**

**Table S1** Data relating to Siberian Crane numbers in natural wetlands of Poyang Lake in the winters of 1999-2016. JXWMB and PLNNR are the abbreviation of the Jiangxi Wildlife Management Bureau and the Poyang Lake National Nature Reserve, respectively. Winter was defined as the year of December.

| Winter | Survey data | Survey organization | Crane number | Reference |
| --- | --- | --- | --- | --- |
| 1999 | 11-15 Nov 1999 | JXWMB | 3,643 | Liu and Jia 2000 |
|  | 26-27 Feb 2000 | PLNNR | 897 | Li et al. 2005 |
| 2000 | Feb 2001 | PLNNR | 3,008 | Wu and Ji 2002 |
|  | Feb 2001 | PLNNR | 2,087 | Wu and Ji 2002 |
|  | 7-11 Jan 2001 | JXWMB | 1,707 | Shan et al. 2012 |
| 2001 | 7-11 Jan 2002 | JXWMB | 3,272 | Shan et al. 2012 |
| 2002 | 7-11 Jan 2003 | JXWMB | 3,800 | Shan et al. 2012 |
| 2003 | Jan-Feb 2004 | PLNNR | 2,760 | Barter et al. 2004 |
|  | 7-11 Jan 2004 | JXWMB | 3,835 | Shan et al. 2012 |
| 2004 | Feb 2005 | PLNNR | 3,131 | Wu et al. 2010 |
|  | Feb 2005 | PLNNR | 2,683 | Liu et al. 2012 |
|  | 7-11 Jan 2005 | JXWMB | 3,004 | Shan et al. 2012 |
| 2005 | 27-31 Dec 2005 | JXWMB | 3,682 | Shan et al. 2012 |
| 2006 | 15-23 Dec 2006 | PLNNR | 2,715 | Liu et al. 2012 |
|  | 27-31 Dec 2006 | JXWMB | 2,587 | Shan et al. 2012 |
| 2007 | 2-5 Jan 2008 | JXWMB | 3,642 | Shan et al. 2012 |
| 2008 | Feb 2009 | PLNNR | 3,008 | Wu et al. 2010 |
|  | 11-15 Feb 2009 | JXWMB | 1,553 | Shan et al. 2012 |
| 2010 | Jan 2011 | PLNNR | 3,374 | Lei et al. 2011 |
|  | Dec 2010 - Feb 2011 | JXWMB | 3,371 | Li et al. 2019 |
| 2011 | 18 Dec 2011 | PLNNR | 4,577 | Li et al. 2012 |
|  | 18-19 Feb 2012 | PLNNR | 3,335 | Zhu et al. 2012 |
|  | Dec 2011 - Feb 2012 | JXWMB | 3,854 | Li et al. 2019 |
| 2012 | 25 Dec 2012 | PLNNR | 1,966 | Liu et al. 2013 |
|  | Dec 2012 - Feb 2013 | JXWMB | 3,224 | Li et al. 2019 |
| 2013 | Dec 2013 | PLNNR | 3,910 | Liao et al. 2018a |
|  | Dec 2013 - Feb 2014 | JXWMB | 3,300 | Li et al. 2019 |
| 2014 | Dec 2014 | PLNNR | 3,872 | Liao et al. 2018b |
|  | Jan 2015 | PLNNR | 3,828 | Liao et al. 2018b |
|  | Dec 2014 - Feb 2015 | JXWMB | 2,957 | Li et al. 2019 |
| 2015 | Jan 2016 | PLNNR | 504 | Liao et al. 2019a |
|  | 18 Dec 2015 | PLNNR | 1,989 | Zhan et al. 2019 |
| 2016 | 18 Dec 2016 | PLNNR | 984 | Liao et al. 2019b |
|  | 18 Jan 2017 | PLNNR | 1,393 | Liao et al. 2019c |
|  | Dec 2016 - Feb 2017 | JXWMB | 1,481 | Li et al. 2019 |

**References**

Barter, M., Chen, L., Cao, L., Lei, G., 2004. Waterbird Survey of the Middle and Lower Yangtze River Floodplain in Late January and Early February 2004. China Forestry Publishing House, Beijing, China.

Lei, G., Lei, J., Tao, X., Hearn, R., 2011. Report on Waterbird Survey of the Middle and Lower Yangtze River in 2011 (Interior report).

Li, F., Ji, W., Zeng, N., Wu, J., Wu, X., Yi, W., Huang, Z., Zhou, F., Barzen, J., Harris, J., 2005. Aerial survey of Siberian Cranes in the Poyang Lake Basin, in: Wang, Q., Li, F., (Eds.), Crane Research in China. Yunnan Education Publishing House, Kunming, China, pp. 27-40.

Li, F., Wu, J., Harris, J., Burnham, J., 2012. Number and distribution of cranes wintering at Poyang Lake, China during 2011–2012. Chinese Birds 3, 180-190.

Li., Y., Qian, F., Silbernagel, J., Larson, H., 2019. Community structure, abundance variation and population trends of waterbirds in relation to water level fluctuation in Poyang Lake. Journal of Great Lakes Research. https://doi.org/10.1016/j.jglr.2019.08.002.

Liao, B., Liu, G., Zhan, H., Liu, F., Wang, S., Zhong, J., Wang, X., 2018a. Quantity and distribution of large-size wintering waterbirds in Poyang Lake, in: Liu, G., Zhan, H., (Eds.), 2013-2014 Survey Reports of Natural Resources at the Poyang Lake National Nature Reserve. Fundan University Press, Shanghai, China, pp. 139-152.

Liao, B., Liu, G., Zhan, H., Yu, D., Liu, F., Wang, S., Zhong, J., Wang, X., 2018b. Quantity and distribution of large-size wintering waterbirds in Poyang Lake, in: Liu, G., Yu, D., (Eds.), 2014-2015 Survey Reports of Natural Resources at the Poyang Lake National Nature Reserve. Fundan University Press, Shanghai, China, pp. 121-137.

Liao, B., Liu, G., Zhan, H., Yu, D., Liu, F., Zhong, J., Wang, X., Yu, C., 2019a. Quantity and distribution of large-size wintering waterbirds in Poyang Lake, in: Liu, G., Luo, H., Zhan, H., (Eds.), 2015-2016 Monitoring Reports on Natural Resources of Jiangxi Poyang Lake National Nature Reserve. Jiangxi Science and Technology Press, Nanchang, China, pp. 134-149.

Liao, B., Hu, X., Zhan, H., Zeng, N., He, S., Liu, D., Zhang, W., Yang, Y., 2019b. Quantity and distribution of wintering waterbirds at Poyang Lake, in: Liu, G., Liao, B., Yu, D., (Eds.), 2016-2017 Monitoring Reports on Natural Resources of Jiangxi Poyang Lake National Nature Reserve. Jiangxi Science and Technology Press, Nanchang, China, pp. 142-151.

Liao, B., Liu, G., Zhan, H., Liu, F., Gong, L., Yu, D., Yu, C., Zhong, J., 2019c. Quantity and distribution of large-size wintering waterbirds in Poyang Lake, in: Liu, G., Liao, B., Yu, D., (Eds.), 2016-2017 Monitoring Reports on Natural Resources of Jiangxi Poyang Lake National Nature Reserve. Jiangxi Science and Technology Press, Nanchang, China, pp. 152-167.

Liu, G., Wu, J., Jin, J., Wen, S., He, S., Cao, R., 2013. Number and distribution of wintering waterbirds at Poyang Lake, in: Liu, G., Jin, J. (Eds.), 2012-2013 Survey Reports of Natural Resources at the Poyang Lake National Nature Reserve. Fudan University Press, Shanghai, China, pp. 110-118. (In Chinese).

Liu, G., Zeng, N., Wu, J., Wen, S., Gao, X., Wang, Y., 2012. Number and distribution of waterbirds determined by ground survey in winter of 2006, in: Li, F., Liu, G., Wu, J., Zeng, N., Harris, J., Jin, J., (Eds.), Ecological Study of Wetlands and Waterbirds at Poyang Lake. Popular Science Press, Beijing, China, pp. 154-162.

Liu, Y.Z., Jia, D.Y., 2000. Report on the distribution of Siberian Cranes at Poyang Lake in November, 1999. China Crane News 4, 4.

Shan, J., Ma, J., Li, Y., Qian, F., Tu, X., 2012. Population and distribution of the Siberian Crane (*Grus leucogeranus*) wintering in the Poyang lakes over the past decade. Zoological Research 33, 355-361.

Wu, J., Ji, W., Liu, G., Wu, X., Gong, L., Wang, S., Gao, Y., Zou, N., Zhan, H., Luo, H., Gao, X., Guo, Y., Wang, X., Yang, Y., 2010. Number and distribution of over-wintering waterbirds in the Poyang Lake by aerial survey. Jiangxi Forestry Science and Technology, 23-28.

Wu, Y., Ji, W., 2002. Study on Jiangxi Poyang Lake National Nature Reserve. China Forest Pulishing House, Beijing, China.

Zhan, H., Zeng, N., Liao, B., He, S., Luo, H., Liu, D., Zuo, Q., Yang, Y., 2019. Quantity and distribution of wintering waterbirds in Poyang Lake, in: Liu, G., Luo, H., Zhan, H., (Eds.), 2015-2016 Monitoring Reports on Natural Resources of Jiangxi Poyang Lake National Nature Reserve. Jiangxi Science and Technology Press, Nanchang, China, pp. 126-133.

Zhu, Q., Zhan, Y., Liu, G., Wu, J., Zhan, H., Huang, Y., Huang, J., Zhang, B., Hu, B., Li, Y., 2012. Investigation of number and distribution of the waterfowl of Poyang Lake in the winter of 2011. Jiangxi Forestry Science and Technology, 1-9.

**Table S2** Data sources and values of tuber density and biomass at three sub-lakes (Dahuchi Lake, Shahu Lake, and Meixihu Lake) of Poyang Lake in the winters of 1999-2016. Winter was defined as the year of December.

| Winter | Tuber density (ind/m^2^) | | | Tuber biomass (g/m^2^) | | | Reference |
| --- | --- | --- | --- | --- | --- | --- | --- |
|  | Dahuchi Lake | Shahu Lake | Meixihu Lake | Dahuchi Lake | Shahu Lake | Meixihu Lake |  |
| 1999 | 4.45 | 17.40 | 11.25 | 1.65 | 5.50 | 5.09 | Wu et al. 2012 |
| 2000 | 10.61 | 11.98 | 2.21 | 3.17 | 1.37 | 0.81 | Wu et al. 2012 |
| 2001 | 6.92 | 12.82 | 9.76 | 1.12 | 3.59 | 5.01 | Wu et al. 2012 |
| 2002 | 43.27 | 8.32 | 13.89 | 27.48 | 4.96 | 8.86 | Wu et al. 2012 |
| 2003 | 134.38 | 14.16 | 5.45 | 16.94 | 5.20 | 3.85 | Wu et al. 2012 |
| 2004 | 5.94 | 3.83 | 8.40 | 2.29 | 1.25 | 2.12 | Wu et al. 2012 |
| 2005 | 2.95 | 37.07 | 2.04 | 1.16 | 14.95 | 0.60 | Wu et al. 2012 |
| 2006 | 0.76 | 16.28 | 9.54 | 0.24 | 17.88 | 1.90 | Wu et al. 2012 |
| 2007 | 0.65 | 13.05 | 0.00 | 0.29 | 3.96 | 0.00 | Wu et al. 2012 |
| 2008 | 2.38 | 13.13 | 14.74 | 0.75 | 11.90 | 9.36 | Wu et al. 2012 |
| 2009 | 5.34 | 5.00 | 13.21 | 0.44 | 0.53 | 1.68 | Wu et al. 2012 |
| 2010 | 0.20 | 0.01 | 0.51 | 0.01 | 0.00 | 0.01 | Gao et al. 2012 |
| 2011 | 5.82 | 15.30 | 21.30 | 0.56 | 1.83 | 3.57 | Huang et al. 2013 |
| 2012 | 0.75 | 0.20 | 0.00 | 1.10 | 0.02 | 0.00 | Cao et al. 2013 |
| 2013 | 22.24 | 0.33 | 1.87 | 0.83 | 0.01 | 0.07 | Yu et al. 2018a |
| 2014 | 9.13 | 1.83 | 8.87 | 0.29 | 0.25 | 0.33 | Yu et al. 2018b |
| 2015 | 0.44 | 0.92 | 0.31 | 0.05 | 0.17 | 0.06 | Yu et al. 2019a |
| 2016 | 5.07 | 0.00 | 1.69 | 0.77 | 0.00 | 0.30 | Yu et al. 2019b |

References

Cao, R., Huang, Y,. Liu, G., Wu, Y., Xiong, H., Liu, D., 2013. Monitoring of *Vallisneria* and their tubers, in: Liu, G., Jin, J. (Eds.), 2012-2013 Survey Reports of Natural Resources at the Poyang Lake National Nature Reserve. Fundan University Press, Shanghai, China, pp. 32-36.

Gao, Y., Huang, Y., Huang, J., 2012. Monitoring of *Vallisneria* and their tubers at Poyang Lake in 2010, in: Zhu, Q., Liu, G., Wu, J. (Eds.), 2010 Survey Reports of Natural Resources at the Poyang Lake National Nature Reserve. Fundan University Press, Shanghai, China, pp. 22-25.

Huang, Y., Liu, G., Wu, J., Jin, J., Xiong, H., 2013. Monitoring of *Vallisneria* and their tubers, in: Zhu, Q., Liu, G., Jin, J., (Eds.), 2011-2012 Survey Reports of Natural Resources at the Poyang Lake National Nature Reserve. Fundan University Press, Shanghai, China, pp. 32-35.

Wu, Y., Ji, W., 2002. Study on Jiangxi Poyang Lake National Nature Reserve. China Forest Pulishing House, Beijing, China.

Yu, D., Gong, L., Yi, W., Xiong, H., Liu, D., Ye, T., Chen, D., Huang, S., 2018a. Monitoring report on *Vallisneria* and winter buds. in: Liu, G., Zhan, H., (Eds.), 2013-2014 Survey Reports of Natural Resources at the Poyang Lake National Nature Reserve. Fundan University Press, Shanghai, China, pp. 37-42.

Yu, D., Luo, H., Wu, Y., Xiong, H., Liu, D., Ye, T., Chen D., Huang, S., 2018b. Monitoring report on *Vallisneria* and winter buds. in: Liu, G., Yu, D., (Eds.), 2014-2015 Survey Reports of Natural Resources at the Poyang Lake National Nature Reserve. Fundan University Press, Shanghai, China, pp. 33-39.

Yu, D., Qi, H., Mei, Y., Huang, C., Xiong, H., Ye, T., Chen, D., He, Y., 2019a. Monitoring on *Vallisneria* and winter buds. in: Liu, G., Luo, H., Zhan, H., (Eds.), 2015-2016 Monitoring Reports on Natural Resources of Jiangxi Poyang Lake National Nature Reserve. Jiangxi Science and Technology Press, Nanchang, China, pp. 37-43.

Yu, D., Qi, H., Liu, G., Mei, Y., Xiong, H., Ye, T., Chen, D., He, Y., 2019b. Monitoring on *Vallisneria* and winter buds. in: 2016-2017 Monitoring Reports on Natural Resources of Jiangxi Poyang Lake National Nature Reserve. Jiangxi Science and Technology Press, Nanchang, China, pp. 39-46.

**Table S3** Number of Landsat 5/7/8 images used in our study in each winter of 1999-2016. Winter was defined as the year of December.

| Winter | Landsat 5 | Landsat 7 | Landsat 8 | Total |
| --- | --- | --- | --- | --- |
| 1999 | 24 | 18 | 0 | 42 |
| 2000 | 28 | 32 | 0 | 60 |
| 2001 | 26 | 33 | 0 | 59 |
| 2002 | 24 | 33 | 0 | 57 |
| 2003 | 30 | 41 | 0 | 71 |
| 2004 | 40 | 28 | 0 | 68 |
| 2005 | 18 | 26 | 0 | 44 |
| 2006 | 30 | 23 | 0 | 53 |
| 2007 | 10 | 30 | 0 | 40 |
| 2008 | 30 | 32 | 0 | 62 |
| 2009 | 27 | 33 | 0 | 60 |
| 2010 | 33 | 37 | 0 | 70 |
| 2011 | 4 | 20 | 0 | 24 |
| 2012 | 0 | 36 | 2 | 38 |
| 2013 | 0 | 36 | 48 | 84 |
| 2014 | 0 | 28 | 31 | 59 |
| 2015 | 0 | 33 | 33 | 66 |
| 2016 | 0 | 32 | 33 | 65 |

**Figure S1** All variables in one figure to show how they are reacting at which time.


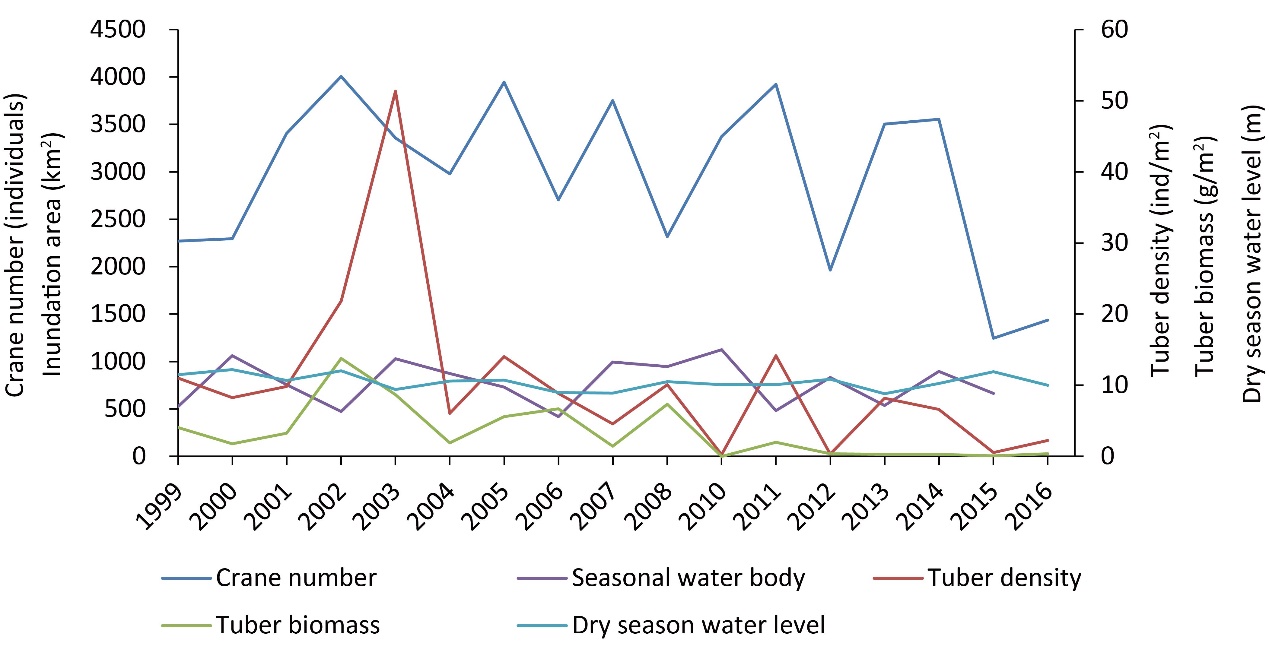


**Figure S2** Permanent water body and seasonal water body of Poyang Lake in the winters of 1999-2016. Winter was defined as the year of December.


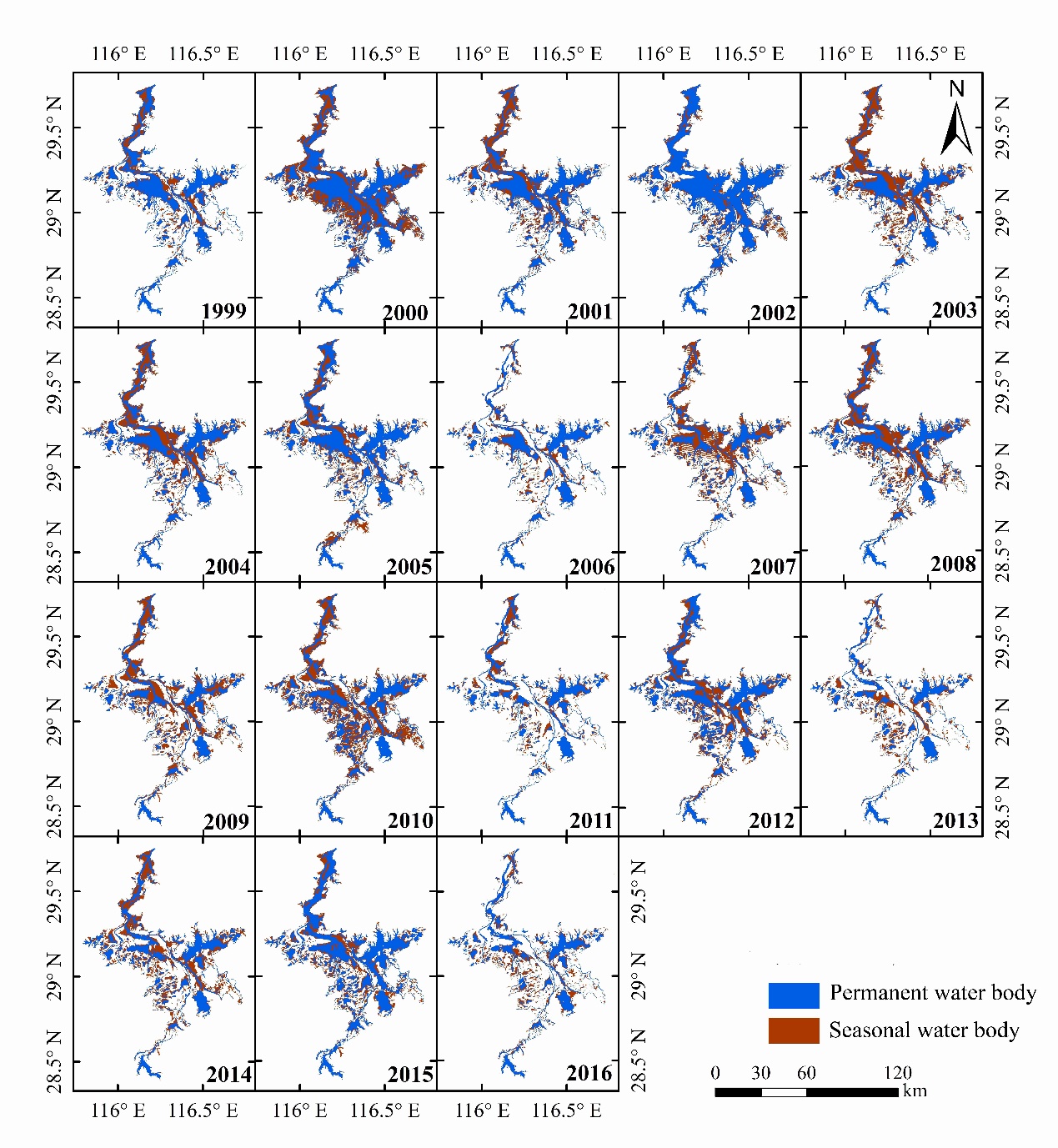

Supplement: Supplementary file 1 — Supplementary Material [file ECE3-10-11055-s001.docx]
